# First person – Anand Singh

**DOI:** 10.1242/bio.050179

**Published:** 2020-01-02

## Abstract

First Person is a series of interviews with the first authors of a selection of papers published in Biology Open, helping early-career researchers promote themselves alongside their papers. Anand Singh is first author on ‘Visualisation of ribosomes in Drosophila axons using Ribo-BiFC’, published in BiO. Anand is a Postdoctoral Fellow in the lab of Saverio Brogna at the University of Birmingham, UK, investigating the role of ribosomes in the nucleus of the eukaryotic cell.


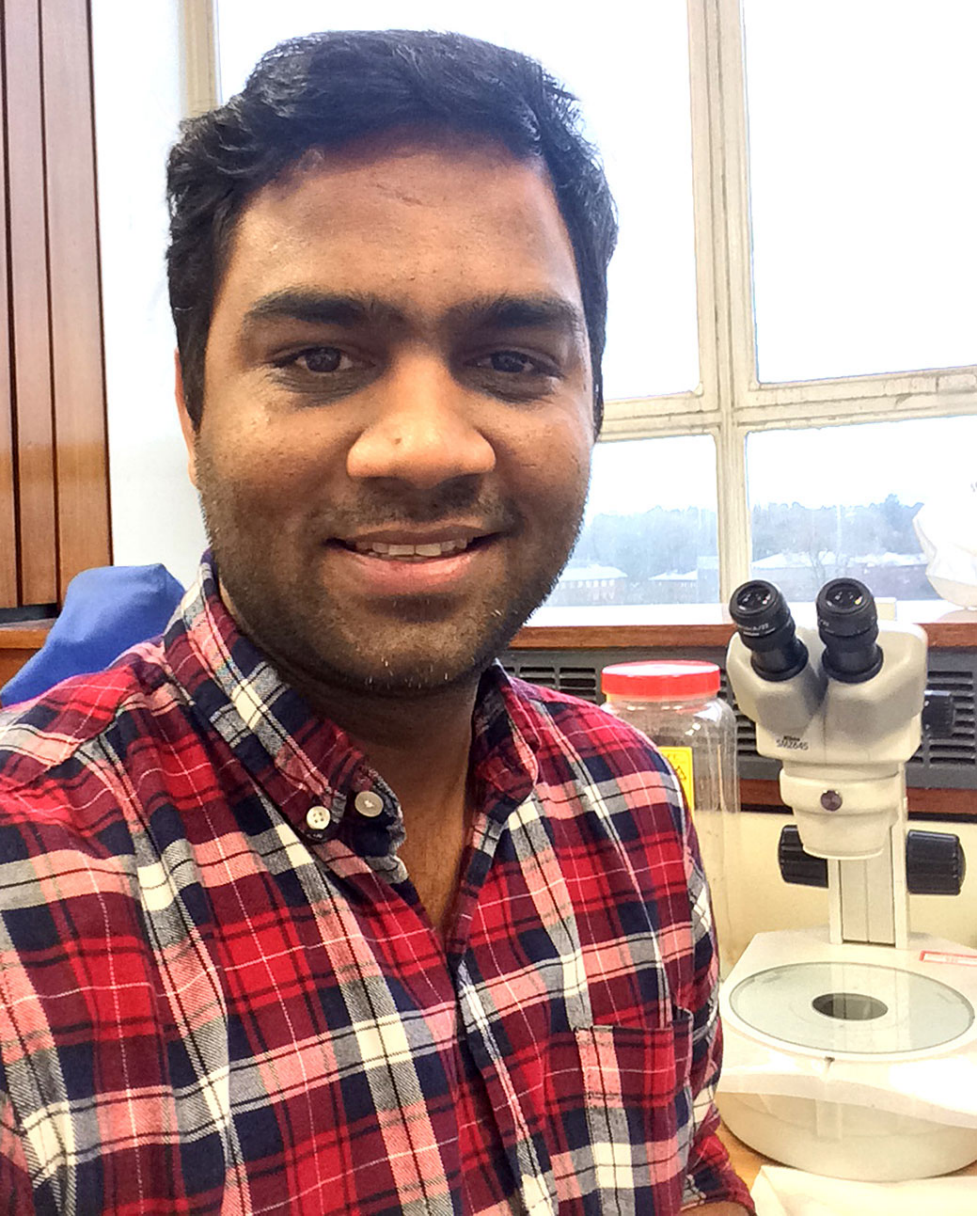


**Anand Singh**

**What is your scientific background and the general focus of your lab?**

I started my research career as a PhD student in the cytogenetics laboratory of Subhash C. Lakhotia at Banaras Hindu University, India. The research interest of my PhD work was to determine the role of *hsrω-n* long non-coding RNA in the regulation of gene expression during heat-shock by using genetic and cell biology approaches. During that study I extensively used the polytene chromosomes of *Drosophila*, a unique experimental system for the visualisation of transcription and RNA processing at gene loci. Subsequently, aiming to further extend my expertise in molecular RNA biology, I joined the laboratory of Saverio Brogna at the University of Birmingham. A long-standing interest of this lab is to understand the mechanism that connects pre-mRNA processing with translation and nonsense-mediated mRNA decay (NMD). As pre-mRNA processing and translation occurs in the nucleus and the cytoplasm, respectively, it has long been understood that NMD, which is strictly dependent on translation, must occur only in the cytoplasm. Contrary to this expectation, we recently published that the NMD core component UPF1 associates with nascent transcripts and starts operating on nuclear mRNAs. Cells depleted of UPF1 show defects in different nuclear processes of gene expression. Presently, we are focusing on understating the role that UPF1 plays in mRNA packaging and export from the nucleus to the cytoplasm.

**How would you explain the main findings of your paper to non-scientific family and friends?**

Proteins are the major building blocks of living organisms and are synthesized by ribosomes, a cellular machine conserved from bacteria to humans. In our paper, we described a novel technique termed Ribo-BiFC that allows straightforward visualisation of ribosomes in different cell compartments. Using Ribo-BiFC we discovered that ribosomes are also present in axons of *Drosophila* neurons. This observation supports the emerging view that ribosomes are present in axons and that some proteins might be locally synthesised in these compartments. Axonal and synaptic local translation might play a central role in neuronal development and memory formation.

“Proteins are the major building blocks of living organisms…”

**What are the potential implications of these results for your field of research?**

Ribo-BiFC is a relatively straightforward technique compared to other recently described methods to visualise ribosomes within cells. By using Ribo-BiFC we found evidence that ribosomes are also present in the axons of *Drosophila* neurons. This method should in principle be extended to other eukaryotic organisms to unveil the factors regulating the ribosome during local protein synthesis in axons of developing as well as mature neurons. We hope that Ribo-BiFC will turn out to be a useful technique to visualise ribosomes in live cells and may change or improve our current understanding of gene expression.

**What has surprised you the most while conducting your research?**

Whilst we observed a good colocalisation of ribosomes and puromycin incorporation (a sign of protein synthesis), some of the ribosomes in the distal region of axons appeared not to be involved in protein synthesis. These may correspond to ribosomes that are either paused on mRNAs after translation initiation or have significantly lower elongation rates. A further investigation on these putative silent ribosomes might unveil a novel mode of protein synthesis regulation in axons.

**What, in your opinion, are some of the greatest achievements in your field and how has this influenced your research?**

Our lab was inspired to develop the Ribo-BiFC technique following reports of high-resolution structures of prokaryotic ribosomal subunits and the revelation that some proteins of the two subunits are adjacent on the assembled ribosome. Following this breakthrough, our group has been interested in visualising ribosomes in living cells by detecting intersubunit contact points microscopically using techniques similar to the Ribo-BiFC.

**Figure:**
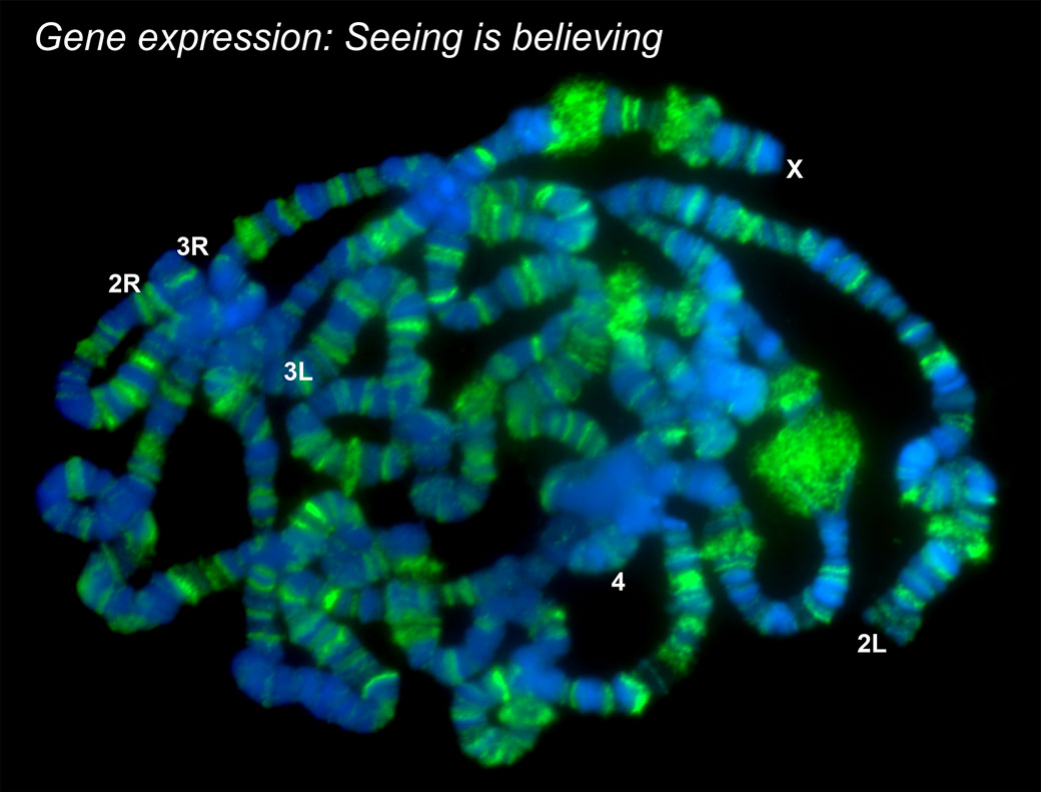
**Microscopy visualisation of transcribing RNA polymerase II (green) on *Drosophila* polytene chromosomes (DNA stained in blue).**

**What changes do you think could improve the professional lives of early-career scientists?**

I believe that quality publications are the foundation for a successful scientific career, as well as for the progress of science. In more than 10 years of my scientific career, I experienced that the most rewarding approach to research is to look at the scientific question from different perspectives. I think it is particularly important to give attention to unexpected results that have often been the most exciting in my research career so far. This approach is intrinsically risky but I think institutions and funding agencies should encourage it. The diversification of the research environment, for example using interdisciplinary approaches, may also help to develop scientific temperament in early-career scientists and promote the ability to work in a team.

“…the most rewarding approach to research is to look at the scientific question from different perspectives.”

**What's next for you?**

I believe that once a researcher, always a researcher. I enjoy using microscopes as well as molecular techniques to study the biology of RNA within the cell. I would like to start my research group in the near future, and currently I am applying for early-career faculty positions and independent fellowships. I find the multiple facets in the complex life of RNAs intriguing and the available cutting-edge techniques that I need to use are inciting rather than daunting.

**Is there anyone you would like to thank?**

I would like to express my heartfelt gratitude to my former and present mentors, scientists who discuss and provide their constructive suggestions to develop my skills and the various funding agencies that help me carry out my work. I am highly thankful to my lovely family and friends for their unconditional support and a constant source of motivation. Special thanks to the Biology Open team for their initiative to promote early-career researchers.
